# Special Hospital Sunday Supplement

**Published:** 1899-06-10

**Authors:** 


### Special Hospital Sunday Supplement.

### The General Hospitals.

It is a curious fact that the general hospitals—the pride and glory of London—are overlooked by a large number of persons in favour of the institutions which possess a distinctive name. For instance, the first man in the street could probably tell anyone who asked him that the Cancer Hospital is located at Brompton, or that the Hospital for Sick Children stands in Great Ormond Street, while it might be necessary to stop half a dozen before the whereabouts of any of the big general hospitals could be ascertained. This is clearly a great disadvantage. All of us, and business people especially, appreciate the vast importance of being known. To be known is to be remembered. And it follows that to be remembered as useful, or faithful, or reliable, is to be remembered with profit. To take but one obvious example, there are many aged ladies and gentlemen without relatives who suddenly call in their lawyer for the purpose of making their wills, and leave the whole question bearing on the purely charitable bequests in the hands of their legal adviser. The names of the institutions have often to be filled in without delay, and those institutions that have been most impressed on the lawyer's mind are those to derive benefit.

It is natural that the well-to-do people, who, owing to the skill of eminent physicians, have been cured or relieved of any special disease, should bestow their thank-offering, or bequeath a legacy, to that hospital which treats the malady from which they suffered. A man having undergone a successful operation for cataract, or a woman whose child has been saved from threatened consumption, would almost as a matter of course sympathise with those who have been in like case, and assist the charity associated with the illness which has been most before their mind, for the public, as a rule, seem to fail to understand that nearly every class of ailment, as well as every kind of accident, is treated at the "generals."

It is the general hospitals that are open both day and night, and where operations may often be found going on at midnight; it is the general hospitals that are first looked to when a huge fire plays havoc with poor human beings; and it is the general hospitals that throw wide open their gates to the thousand and one casualties which are constantly occurring, cases that vary between a workman's fall from a lofty scaffold and a cyclist's nasty spill, or between an all-but-successful suicide and a wound resulting from a drunken fight.

At a recent meeting at the Mansion House a speaker, advocating the cause of a hospital which stands in close proximity to the busy railway termini in Euston Road, said that it was "the great railway hospital of London." "How very few," he added, "of the immense army of railway travellers ever give a thought, much less a donation, to the very institution to which they would in all probability be conveyed were the train in which they speed along at the rate of sixty miles an hour to come to grief while within reasonable distance of this end of their journey." If only for their constant readiness to meet emergencies, the debt that the general public owes to our admirable general hospitals is immense.

One has only to pay a visit to any of the general hospitals to learn how tremendous is the need they serve. Take a peep into the casualty room as a commencement. There you will observe everything is in apple-pie order; there are seats and couches and bandages and lotions all set ready for immediate use and for any emergency. Nurses, with gentle hands, are calmly awaiting whatever may turn up. If you are able to remain for a time you will in a few hours be a witness of the results of as many accidents and seizures as you would previously have imagined would occur in as many weeks. Your eyes would be opened once for all, and you would, we doubt not, straightway resolve to contribute to your utmost to the Hospital Sunday Fund. A tour of the medical and surgical wards, in which may be found all manners of strange cases, would prove a lesson that few, if any, could withstand.

No one ought ever to consider that the general hospitals are outside the circle of direct claim upon them. Perhaps a true incident will suffice to bring home the full force of this statement.

A barrister who for some 30 years had safely travelled to and from his office in one of the Gray's Inn squares was one day during 1898 knocked down by a passing cab. He was picked up and taken to a general hospital which he had passed and repassed daily during all the years he had been in practice. The house surgeon on duty examined him, and it was discovered that he had sustained a fractured leg in addition to a number of more or less severe bruises. It was, of course, necessary to place him in a bed in one of the wards, and there he remained until he was well enough to be removed to his own home in the suburbs. A few days after his removal the secretary of the hospital received a cheque for a handsome amount from him, and with the gift a letter in which he stated that until his personal experience of the excellent work done, and the real need supplied, by that general hospital he had not thought it necessary to subscribe regularly to its funds. He had, however, made up his mind that in the future one of the first calls upon his charity should be the whole of the general hospitals of London. The barrister referred to is now one of the active members of the committee of management of the hospital to which he was carried.

If individuals would only reflect that the next day or even the next hour might find them the victims of a street accident—for accidents happen to the careful folk who always keep to the pavement as well as to the venturesome on the dangerous crossings—and that they might, even without their consent, be taken to one of the general hospitals, they surely would give so freely that the state of the funds of all our deserving centres of medical and nursing skill would be very different from what they are.

Of course it says much for the generosity of mankind that so many institutions are already kept going by voluntary offerings, but how much more beneficent the work would be if the secretaries and other officials of the various institutions were able to devote all their energies to organisation and supervision instead of to appeal writing and appeal devising!

### The Special Hospitals.

After the general hospitals come the special ones, and it is in regard to these that the Hospital Sunday Fund more particularly deserves the support of the public. Every giver, whether he puts a shilling on the collecting plate, or sends a cheque for thousands to the secretary, knows that he is right when he gives to one or other of the great general hospitals of London. Their work is so immense, and the suffering with which they endeavour to cope is so appalling, that whatever abuse may be alleged to exist here and there it is swamped and lost in the midst of the untold benefits which, as is admitted on every hand, these institutions pour out ungrudgingly upon the halt and maimed and blind and generally wretched people who throng their waiting-rooms, and endeavour to gain admission to their wards. Everyone feels, then, that to give, and to give freely, to the great hospitals is always good. The same, however, cannot always be said in regard to the specials. A feeling of doubt has got abroad, questions have been raised as to this and that special hospital, and all such questionings afford excuses for buttoning up the pockets when men are asked to give. Nor can it be admitted that this is always a mere matter of stinginess. We appeal to everyone to contribute to the hospitals; but we quite understand the feelings of those who, when they have doubts about the object, hesitate to contribute. No one has a right to look upon his neighbour as niggardly or tight-fisted merely because he declines to hand out his cash when asked for it in the name of charity. It is a perfectly proper and business-like instinct which leads men, even when their desire is to give freely, to demand some sort of guarantee that the object to which they give is good. And this is where the Hospital Sunday Fund does such great service, not only to the hospitals of London, but to those who with liberal intentions are still held back by doubt and by natural English hatred of being taken in; for the Hospital Sunday Fund gives just the sort of guarantee which the cautious giver wants. It gives the guarantee of an independent inquiry into the work and the conduct of the various hospitals among which its funds are distributed, so that everyone who gives his shilling or his cheque on Hospital Sunday knows that his money goes to hospitals whose work has been done under supervision, and that, so far as can be secured by the anxious care of a committee fully representing all the interests concerned, the Fund collected is distributed according to the *bonâ fide* wants of the various recipients. This is a great thing, and from the donor's point of view the most important thing, namely, that by giving through the Hospital Sunday Fund an assurance is received that the gift is well expended; and, as we have already said, this applies more especially to special hospitals, for it is especially in regard to them that gifts have been held back by doubt. Yet when we remember the splendid services which specialism has rendered to medicine and mankind, and recall that it has been in special hospitals and in the special departments which are now attached to all our great general hospitals, that some of the greatest advances in treatment have been worked out, so as to have become the common property of the medical profession throughout the world available for the benefit of mankind at large, we must recognise the claims of specialism in medicine, and also the claims of the special hospitals and special departments to that monetary support by aid of which alone they are enabled to carry on their good work; and one of the great advantages of Hospital Sunday is that Hospital Sunday stands as an intermediary between a liberal but not always well-informed public and that crowd of special charities whose very multitude is apt to cause confusion, and perhaps to breed indifference in the public mind.

With the courteous assistance of the secretaries of a number of the special hospitals we place before our readers, in a brief form, the special claims on which some of the most representative of these institutions base their appeal to the public for support:—

### ACCIDENTS.

Although accidents are treated at all the general hospitals, there is a large institution in the East End which is specially reserved for that purpose. It is situated among a teeming population of poor hard working people, in a district which is fitly described as both the "workshop" and the "port" of London. Accidents are treated here at the rate of five an hour for every day of every year; which means that five miles of men standing side by side are treated for accidents only in 12 months. Another strong claim of this charity is that working men themselves give £600 a year in subscriptions and donations towards it. There is no endowment, and if subscriptions fall off, the work has to be curtailed. The hospital is free to all; no letters are required.

### CONSUMPTION AND DISEASES OF THE CHEST.

Much has been heard of late about tuberculosis, concerning the control and the prevention of which a Congress has just been held in Berlin—a peaceful meeting of the Great Powers to organise concerted action against a common enemy which kills more victims than any war which we are likely, let us hope, to see in our time. But many and various as are the forms which tuberculosis takes on, the mischief centres mainly around consumption or tuberculosis of the lungs. People may die of tuberculosis in any form, but it is by the consumptive that the disease is spread. It is the consumptive workman in the factory, the consumptive clerk in the office, and especially the advanced consumptive living out the remainder of his life in a poverty-stricken home, by whom the disease is passed on to others. As a mere matter of self-protection, then, the treatment of consumptives in special hospitals is good policy. In fact, it may properly be urged that whatever claims special hospitals for consumption may have had upon the public in the past, they have double claims now, in that we now know that for every case that receives the benefit of treatment within their walls, the public receives the benefit of being relieved for the time of one source at least from which the disease might spread through the community. But putting policy on one side, consumption has a special claim upon the purses of the charitable, if only from mere pity; for consumption is all too apt to attack people who are just entering upon the active work of life, and are just taking upon themselves responsibilities which cannot be cast aside. Consumption is a most expensive disease. Even those who, under ordinary circumstances, are well-to-do find when one of their relatives is attacked with consumption what a drain upon the family resources the proper treatment of such a case entails; and among the poor the drain is so great that the sick man has almost inevitably to go to the wall—unless, indeed, kind charity steps in and gives the man a chance. Seldom, indeed, does charity do a better thing than when she intervenes and restores to health the young bread winner who has been struck down by early phthisis.

The most indefeasible of the many claims of the great hospital at Brompton for patients suffering from consumption to the support of the public is that in 1898 no less than 1,804 in-patients were admitted, of which 1,514 were discharged, many having been greatly benefited by the treatment. The average stay of each patient was 69 days, and the daily average number of beds occupied was 274. Moreover, 13,988 new cases received medical treatment and medicine as outpatients, while 74,338 attendances were registered. The hospital contains 321 beds, and its yearly requirements are estimated at a minimum of £25,000 a year. Beyond this, assistance is wanted for two admirable purposes. One is the home for private and staff nurses, which is now approaching completion, and the other is a country branch and convalescent home. The latter has been decided upon in consequence of the unanimous and urgent advice of the medical staff, and it is estimated that the site and building will cost £20,000. On the recommendation of the medical committee, the open-air treatment is to be made available in a portion of the hospital.

In the North of London, at a hospital situated 400 ft. above the sea-level, the open-air treatment of consumption is already carried out, and a specially-designed structure to accommodate 40 patients undergoing this treatment is immediately to be erected in the grounds at a cost of £2,000. The hospital contains 80 beds, and there are at least 12 applicants for every vacant bed. Children are admitted from three years of age upwards, and there is a Samaritan Fund for the assistance of the most needy cases. Last year 3,502 out-patients and 460 in-patients were treated. The majority left the hospital to return to their respective occupations greatly improved in health. The death-rate was 4·8 per cent. All patients received are seen by the House Committee on entering, and inquiry made as to their circumstances before they are permitted to receive free treatment. The support of the public is particularly claimed in the endeavour of the hospital to make the open-air treatment a success.

The East End has also a very important hospital for patients suffering from diseases of the chest, which was established in 1848. The number of in-patients treated yearly is nearly 1,000, and of out-patients 15,000. This number would be greatly increased if funds permitted, but owing to the lack of them several wards have had to be entirely closed. This all the more sad because many of the cases waiting admission are of the most distressing character.

The oldest hospital of this kind, established in 1814, is threatened with a similar calamity. Beds are reserved in this institution for the immediate reception of out-patients, who are found to be suffering from acute and dangerous diseases of the chest of recent origin, and who cannot be properly treated unless they are at once admitted as in-patients. There are also five cots for the reception of children suffering with acute bronchitis, &c. But the income has fallen off so seriously that unless £2,000 is received between now and the end of June one of the three large wards containing 30 beds will have to be closed. The pity of it! That great congresses should be held and talk should flow about the prevention of consumption, and yet that we cannot keep open the wards which we possess for the treatment of its victims!

### CANCER.

On behalf of the single charity for the exclusive benefit of patients suffering from this dreadful disease the first claim it makes on the support of the public is that it is absolutely free. Another is that beds are set aside for life cases. Moreover, owing to the nature of the disease, a most liberal and expensive dietary has to be provided for both patients and nurses, and the dressings used far exceed both in quality and quantity those necessary at a general hospital. A special feature is made of bright, cheerful, and attractive surroundings, so as to do as much as possible to draw the thoughts of the patients from their sufferings, which, in many cases, are as keen mentally as physically.

### CHILDREN.

Perhaps it may seem unnecessary to say a word in favour of the great claims which special hospitals for children have upon the continued support of the public. Love of children and sympathy for children in distress are so generally felt and often so loudly expressed that it is difficult to believe, although still it is the fact, that hospitals for these little ones are actually in want of money. It surely is not creditable to this great London in which we live that children full of that vitality and power of recovery which is inherent in youth and by aid of which they ought quickly to get well should be allowed to fade away in garrets, when by removal to the wards of a children's hospital they could almost certainly be restored to health, and helped to grow up to a self-supporting manhood. Yet such is the fact, and all for the sake of a few pounds! In asking people to give, and to give freely to the Hospital Sunday Fund, we feel that one great claim upon them is that a portion of the fund goes to the assistance of the children's hospitals.

The oldest and largest children's hospital in the British Empire points to its record last year as its primary claim on the liberality of the community. No less than 2,067 in-patients were treated in the wards; 238 patients were sent to the convalescent branch; and 24,249 new out-patients were registered in the out-patient department. In other words, 26,554 sick children were treated during the year. The average annual expenditure of the charity is £15,000, of which £8,000 has to be raised every year to keep the wards open. A debt of £20,000 has lately been incurred in the purchase of the adjoining hospital, which has secured for the little sufferers a garden of half an acre, enabled the committee to open a whooping-cough ward, and to provide proper accommodation (urgently needed) for the nursing staff.

The second largest hospital for children claims support on account of the growing demand for treatment in the East-end of the metropolis. Last winter a seaside branch was opened, and this has already been found to be of the greatest advantage to the hospital. As soon as patients are well enough to move they are drafted to the seaside, where they have a better chance of making a complete recovery, while beds in the hospital are thus left free for the reception of more acute cases. The cost of maintaining the branch will involve an expenditure of £700 a year, towards which, up to the present time, only about £50 has been guaranteed by annual subscriptions. As to the hospital itself, the cost of maintenance averages £8,000 a year, of which the committee have to rely upon donations and legacies for about £5,800.

In the North-east a large hospital, founded in 1867, ministers especially to the districts of Shoreditch, Bethnal Green, and Hackney, with a population of nearly half a million, and is also required to undertake the special work devolving on a children's hospital in a much wider area, comprising Old Ford, Bow, Whitechapel, Spitalfields, and the outlying districts of Tottenham, Walthamstow, and Leytonstone. There are at present only 57 beds, and it is proposed to double the number at an estimated cost of at least £25,000. Although the committee have as yet only some £6,000 in hand, the need for increased accommodation in every department is so pressing that they intend to commence building in the autumn. The hospital is lighted by electricity, and possesses an exceptionally powerful Röntgen rays apparatus, from which important results are being obtained. Particular attention is given to ophthalmic work, and there is an efficient and well-organised dental department in which the practice of dentistry in all its branches applicable to children is undertaken. A carefully-devised system of inquiry into the circumstances of out-patients was commenced in June, 1898, and although every new case now comes under the scrutiny of the inquiry officer, acting under the control of the House Committee, there has not been the slightest friction between the parents and the officials. The most careful investigations have, however, shown that very little abuse of the charity is attempted, not more than one case in 800 being found unsuitable. The hospital is well supported in the immediate neighbourhood, but as its average expenditure is £5,700—to be very largely increased when the enlargement scheme is completed—and the annual subscriptions only amount to about £1,500, there is ample scope for people to help those who are helping themselves.

There are several other children's hospitals in London (we do not pretend to enumerate all the special hospitals either of this or any other kind), but what are they even altogether among the enormous multitude of children who are growing up in our midst every year? Growing up straight or crooked, strong or weak, healthy or diseased, according to the bent given to them in their childhood. The responsibility which rests upon a community which, for want of a little hospital treatment during childhood, allows disease to remain implanted on its rising generation is serious indeed.

### DENTAL.

The advantages of dental hospitals were never more obvious than they are in the present day, when the hurry and bustle of life tempt so many people to take their meals in a hurry. Moreover, it is generally recognised that teeth are not so strong as they used to be, and in many cases need to be constantly looked after. The hospital founded in 1858 has been enlarged to its utmost extent, and being still inadequate, both in accommodation and sanitation, for the ever-increasing number of patients who avail themselves of its benefits, it has become absolutely necessary, if the work is to be carried on efficiently, to build a new hospital. In fact, the building has been begun and it is hoped will be completed in about two years. But to place the building fund in a satisfactory position, a sum of £3,000 is required this year. As evidence of the growing needs of the public for treatment, it may be mentioned that while the number of cases admitted in 1874 was 19,255, the number admitted last year was 63,298.

The committee of another of these institutions, at which only the necessitous poor are received, a subscriber's letter entitling a patient to a free administration of gas, also find that each year brings an increase in the number of patients treated, but that there is no substantial increase in the number of subscribers. It is felt that with such an excellent and well-appointed building as they possess, so much more could be done to relieve distress if only they had more funds at their disposal. Within the last six months a new mechanical department for the supply of artificial teeth has been started which has taxed the resources of the hospital to the utmost, as the number of patients in this department is more than the present staff are able to cope with. The committee would be able, with an increase in settled income, to consider the possibility of providing salaried assistance for dealing with some of the large proportion of applicants who, in existing circumstances, cannot obtain relief.

### FEVER.

Since 1802 the hospital which is intended especially for people suffering from scarlet fever and diphtheria has admitted 85,000 patients. It is a reasonable deduction that the institution has therefore been the means of preserving many times eighty-five thousand persons from infection. It provides isolation and treatment for those who do not use the general hospitals in case of ordinary sickness and decline to seek admission to the fever hospitals which are supported by the rates, even when their homes are invaded by a dangerous and often deadly malady. The patients are required to pay a fee equal to about a fourth of their cost, the other three-fourths being defrayed by the institution. The fee is fixed at this low figure in order to induce individuals to seek admission rather than remain for treatment in their own dwellings, where they can hardly fail to be a source of peril to all around them and to the public at large. At this hospital some of the most important discoveries of modern times relating to the nature and treatment of infectious fevers have been made, and it is claimed that much life-prolonging knowledge is from time to time acquired for the benefit of the community. The domestic servants of the governors, and certain employés of subscribing firms, clubs, and hotels, are treated free of all charge. For all funds, save the small amount contributed by some of the patients, the hospital depends upon voluntary help.

### LYING-IN.

The most prominent of these admirable institutions was founded in 1752, and has since that time provided for the delivery of upwards of 100,000 poor women. More than 1,100 patients are admitted every year, and a similar number are treated by the staff in their own homes. This is the only charity which receives deserving single women with their first child. Attached to it is a large and important training school, and nearly 250 medical pupils, midwives, and monthly nurses are annually received for training. The importance of this branch of the work is obvious. In order to meet the greatly increased demands on the hospital, an extension is being carried out, and a new nurses' home has been built, and will shortly be opened. Upwards of £7,000 is still needed to enable the committee to discharge their liabilities without trenching upon the very small amount of invested funds.

### OPHTHALMIC.

The utility of one of these hospitals is demonstrated by the amount of relief it affords to the suffering poor. All patients are admitted free, and without letter of recommendation. The only credential requisite is, in short, defective sight, combined with poverty. The number of in-patients in the year is nearly 2,000, and that of out-patients upwards of 25,000. Its annual expenditure is about £8,000, and its reliable income from annual subscriptions and invested funds is only £1,500. Just now money for furnishing the new building about to be opened is urgently wanted, as well as help to meet current expenses.

Another ophthalmic hospital, founded in 1843, has also outgrown its accommodation, and strenuous efforts are being made to obtain funds for its enlargement and gradual reconstruction, a sum of over £2,000 having already been subscribed for the purpose. The advantages to the poor have been amply proved by the number who have had blindness averted, vision restored, disfigurement removed, and disease alleviated. About 400,000 patients of both sexes and every age from all parts of the United Kingdom and colonies have received treatment since its foundation, the number of attendances last year being 30,029. Situated in a densely-populated part of the metropolis, many of whose inhabitants are employed in watch and clock making, working in jewellery, type-founding, printing, metal engraving, and other occupations in which accidents and diseases of the eye are very prevalent, it has likewise been of great service to business employés, governesses, school teachers, clerks, and domestic servants.

On behalf of one of these institutions it is claimed that, founded in 1816, it has rendered good service to a dense population of workers in its immediate vicinity, whose means of livelihood would have been destroyed by loss of, or injury to sight. It also relieves many patients from the suburbs and the country, where no eye hospital exists to meet their wants. In 1898 there were 10,809 out-patients and 585 in-patients. A new building upon a larger site is urgently required. Here are examples of recent cases: A child aged 13, who was admitted from one of the industrial schools early in May, practically blind at the time of admission from the intense inflammation of both eyes. She is now able to go about by herself, and has every chance of good vision. A child aged 2, of very poor parents, would undoubtedly have lost the sight of both eyes by acute septic inflammation, but as the result of a month's constant treatment in the hospital—every hour, night and day—her vision is now practically perfect. There are numerous cataract patients who come in perfectly blind, but after operation leave with enough vision to do their daily work. Ophthalmia neonatorum nearly always ends in total blindness, unless under constant treatment, which, in the poorer classes can only be obtained at a hospital, and these cases are extremely numerous.

### ORTHOPÆDIC.

The institution founded in 1838 has, since its opening, treated over 80,000 patients. There are 50 beds, and a feature of this class of hospital is that the average duration of the treatment of the cases is about 100 days. Moreover, nearly 60 per cent. of the cases are from the provinces, and as it is impossible to have them under supervision after they leave the hospital, they are kept as long as is necessary. The patients include many farm labourers, dressmakers, shop assistants, and domestic servants, male and female cases being about equal in number. Last year there were 180 in-patients and 726 out-patients. Seventy-four were discharged as cured, and 110 as improved. More room, a better building, more light and air, and more beds are needed. At present, patients who are told to come to the hospital immediately for treatment may have months to wait for admission. The infants' ward of ten cots is always filled.

At another of these hospitals for the treatment of diseases of the spine and joints, contractions and distortions of the limbs, the attendance of patients average over 10,000 yearly. It is absolutely free, the only qualification for admission being that the patients are unable to pay for surgical treatment. Each patient receives the continued attention of the surgeon for a considerable length of time, and a much longer residence of the in-patients suffering from spinal and joint disease is necessary than is compatible with the work of a general hospital. The surgeons give their individual attention to the construction of all the appliances which they use in treatment. The hospital has long required partial rebuilding for various reasons, but chiefly on account of necessary sanitary alterations. Having at length to choose between commencing structural alterations and shutting up the building until the requisite funds came to hand, the committee wisely adopted the former alternative, relying upon the generosity of the public to help them out of their difficulty. A great number of the patients can be entirely cured, and others can be so far improved as to be relieved from pain and enabled to get about and earn their living, instead of remaining helpless and burdens to themselves and others.

### PARALYSED AND EPILEPTIC.

A great hospital for the paralysed and epileptic claims that it is the single institution established to treat brain diseases, and thus proves a school for their study and alleviation, without which the sufferings and risks of many a brain-worker would be far greater than they are at present. It relieves sufferers unfitted for general hospitals; it deals with maladies among the most mysterious and terrible which afflict humanity; it is the source of a beneficent knowledge which is helping to assuage pain and to save life in all parts of the civilised world; and the demands upon it increase yearly, and are already far beyond its resources. To emphasise these claims it may be added that up to December 31st, 1898, more than 1,700 different cities, towns, and villages of the Empire have been represented by from one to a hundred patients each, exclusive of London; that the types of cases treated in the wards last year included right and left-sided paralysis, double paralysis, epileptic convulsions, neuralgia, inflammation of nerves, St. Vitus's dance, insomnia, wry-neck, contractions, apoplexy, head jerks, hysteria, atrophy, shaking palsy, inflammation of brain, inflammation of spinal cord, lockjaw, cancerous and other tumours pressing upon the brain, and spinal cord, involving paralysis, blindness, or convulsions; that the oldest patient admitted in the year was 78 years, and the youngest six weeks; and that the larger proportion of sufferers treated in the free as well as in the "contributing" wards are of the educated classes, although all alike are in needy circumstances.

For paralysed children and those suffering from nervous diseases there is an institution in the West End, with fifty beds available. Boys are admitted up to the age of ten, and girls up to that of 12. Nearly all the beds of the hospital are free, and there is always a long list of applicants waiting their turn for admission, especially in winter. The institution is well equipped with scientific apparatus and special appliances for the treatment of diseases of the nervous system. The payments made do not amount to a fourth of the expenditure.

### SEAMEN'S.

The celebrated hospital for sailors has lately been before the public in a prominent manner owing to the establishment, at the instance of Mr. Chamberlain, of the London School of Tropical Medicine, in connection with its branch in the Royal Victoria and Albert Docks. But though Her Majesty's Government has contributed towards the new building, and the public have so far come forward generously by contributing the greater portion of the money required to build and equip the new school, further funds have still to be raised in order to maintain the school and the new wing which it has been found necessary to add to the hospital. The latter is to be enlarged to 45 beds, owing to the many urgent cases which are daily brought in. In the last 20 years the progress of the Seamen's Hospital Society has been remarkable. In 1898 over 25,000 sick and injured seamen and others were treated within the wards and in the out-patients' departments of the two hospitals and dispensaries as against 8,162 10 years ago, and 4,563 in 1898. Among the patients there have always been every year a large number of Europeans, as well as Asiatics, Africans, and natives of British and other colonies, suffering from tropical disease, and there is no other institution in the country where such cases are to be found in a like number and variety. Another feature of the hospital is that arrangements have been made for fever nurses from the Colonial Nursing Association to receive training in tropical medicine before proceeding to their destinations abroad.

(*For Hospitals for the Throat and Ear, and for Women's Hospitals, see page* 20.)

### HOSPITAL SUNDAY.

Now we have come to the end of our space, although it can by no means be said that we have come to the end of what could be said, and perhaps ought to be said, about the incalculable amount of good that is being done every day by those medical charities in support of which collections will be made on Hospital Sunday. We have given in tabular form a list of these institutions, showing their income and expenditure, and pointing out, as far as it is possible so to do by mere figures, what is the character and extent of the work which these great establishments, "supported by voluntary contributions," are carrying out, and we have entered somewhat more particularly into the special claims which certain classes of hospitals have upon the support of the public. This we have done partly in order that those who are charitably minded may see how vast is the field and how great is the necessity for the assistance which we hope they will pour out without stint on Hospital Sunday, and partly in order that those who are careless and indifferent may, if they chance to study the facts which we have put together, be driven of very shame to contribute to the relief of the suffering men, and women, and children, who live along with them as fellow citizens, almost as neighbours, in this great city, persons whose lot when sickness comes upon them would be too sad for words were it not for the existence of the hospitals, for which in the name of Hospital Sunday—that great link between religionists of every creed—we now ask for help.

It is not much that is asked. Large donations are good, subscriptions are good, even legacies are good, although not so good, in fact, as some imagine, for they too often mean the loss of some good friend, whose help and support will be missed in many ways. But what is specially asked for on Hospital Sunday is something different. It is that open, free-handed giving which everyone some time or another in his life indulges in, and to which, be it said, he will some time or another look back with comfort and satisfaction. On Sunday, in every pulpit, people will be asked to contribute to the hospitals, and many as are the creeds and varied as are the formularies in accordance with which God is worshipped in our midst, it will be found that every creed and every formulary will allow alms for the hospitals to be asked for in the name of religion. On this broad platform of charity all faiths unite. Let us hope that all will vie with each other in well-doing.

### Metropolitan Hospital Sunday Fund, 1899.

### A YEAR'S WORK IN THE HOSPITALS AND MEDICAL CHARITIES OF LONDON.

### NEWINGTON AND SOUTH DISTRICT.

Comprising Battersea, Wandsworth, Tooting, Balham, Streatham, Brixton, Lambeth, Newington, Southwark, Bermondsey, Camberwell, Greenwich, Deptford, Lewisham, Blackheath, Woolwich, &c.

| No. of Beds. | No. of Beds Daily Occupied. | HOSPITALS. | In-patnts. | Out-patients. | Total Expenditure. | Income: Charitable. | Income: Proprietary. | Income: Patnts' pymnts. | Total Income. | Legacies not included in previous column. |
|---|---|---|---|---|---|---|---|---|---|---|
| | | | | | £ | £ | £ | £ | £ | £ |
| 650 | 424 | Guy's ... | 6,289 | 89,533 | 66,274 | 24,602 | 30,136 | 4,103 | 58,841 | 13,476 |
| 25 | 21 | Miller ... | 292 | 18,118 | 3,508 | 2,564 | 338 | ... | 2,902 | 2,150 |
| 248 | 205 | Seamen's ... | 2,548 | 22,850 | 18,776 | 8,001 | 4,263 | 447 | 12,711 | 3,026 |
| 570 | 407 | St. Thomas's ... | 6,079 | 64,975 | 52,653 | 4,316 | 41,474 | 358 | 46,748 | 5,293 |
| 66 | 54 | Evelina, for Children ... | 949 | 7,633 | 5,552 | 3,945 | 645 | 83 | 4,473 | 2,175 |
| 57 | 48 | Home for Sick Children ... | 214 | 1,353 | 1,665 | 1,118 | 235 | 305 | 1,658 | ... |
| 24 | 21 | General Lying-in ... | 546 | 1,791 | 3,413 | 764 | 2,660 | ... | 3,424 | ... |
| 36 | 15 | Clapham Maternity and Dispensary ... | 331 | 5,870 | 2,226 | 346 | 926 | 804 | 2,076 | ... |
| 52 | 50 | Royal, for Children and Women ... | 599 | 8,777 | 4,045 | 3,296 | 929 | 242 | 4,467 | 4,000 |
| 40 | 26 | Royal Eye ... | 433 | 16,685 | 3,034 | 1,865 | 107 | 518 | 2,490 | 250 |
| 10 | 2 | Hospital for Diseases of the Skin ... | 26 | 4,236 | 1,024 | 205 | 178 | 547 | 930 | ... |
| 396 | 225 | Metropolitan Convalescent ... | 3,407 | ... | 7,714 | 6,031 | 337 | 140 | 6,508 | 9,890 |
| 10 | 5 | Phillips' Memorial Homœopathic ... | 77 | 1,000 | 575 | 350 | ... | 204 | 554 | ... |
| 11 | 5 | Eltham Cottage ... | 62 | ... | 375 | 228 | 11 | 83 | 322 | ... |
| 20 | 10 | Beckenham Cottage ... | 152 | ... | 600 | 581 | 22 | 103 | 706 | ... |
| 18 | 10 | Blackheath Cottage ... | 142 | 1,121 | 795 | 956 | 22 | 103 | 1,081 | 100 |
| 32 | 21 | Bromley Cottage ... | 323 | ... | 1,284 | 867 | 103 | 185 | 1,155 | 100 |
| 14 | 10 | Chislehurst, &c., Cottage ... | 148 | ... | 608 | 410 | 11 | 136 | 557 | ... |
| 12 | 7 | Sidcup Cottage ... | 97 | 43 | 491 | 459 | 20 | 136 | 615 | ... |
| 7 | 6 | Shortlands Convalescent ... | 113 | ... | 1 90 | 155 | 1 | 5 | 161 | ... |
| 16 | 13 | Livingstone Cottage ... | 143 | ... | 778 | 659 | 3 | 66 | 728 | ... |
| 2,314 | 1,585 | | 22,970 | 243,985 | 175,580 | 61,718 | 82,421 | 8,568 | 152,707 | 40,460 |
| | | DISPENSARIES. | | | | | | | | |
| ... | ... | Battersea Provident ... | ... | 20,732 | 3,210 | 79 | 61 | 3,104 | 3,244 | ... |
| ... | ... | Brixton, &c. ... | ... | 4,246 | 607 | 519 | 27 | 70 | 616 | 200 |
| ... | ... | Camberwell Provident ... | ... | 10,308 | 1,763 | 333 | 120 | 1,527 | 1,980 | ... |
| ... | ... | Clapham ... | ... | 1,507 | 329 | 288 | 7 | 121 | 416 | 183 |
| ... | ... | Deptford Medical Mission ... | ... | 2,311 | 409 | 344 | 43 | 67 | 454 | ... |
| ... | ... | East Dulwich Provident ... | ... | 3,372 | 791 | 96 | 1 | 702 | 799 | ... |
| ... | ... | Forest Hill ... | ... | 2,346 | 751 | 240 | 10 | 508 | 758 | ... |
| ... | ... | Royal South London ... | ... | 6,146 | 635 | 554 | 114 | ... | 668 | ... |
| ... | ... | South Lambeth, &c. ... | ... | 2,273 | 588 | 430 | 15 | 183 | 628 | 10 |
| ... | ... | Walworth Provident ... | ... | 688 | 237 | 8 | 36 | 146 | 190 | ... |
| ... | ... | Wandsworth Common ... | ... | 893 | 212 | 46 | ... | 166 | 212 | ... |
| ... | ... | Woolwich, &c., Provident ... | ... | 1,183 | 293 | 23 | 13 | 194 | 230 | ... |
| 2,314 | 1,585 | | 22,970 | 299,990 | 185,405 | 64,678 | 82,868 | 15,356 | 162,902 | 40,853 |

### CITY AND EAST CENTRAL DISTRICT.

Comprising the City, St. Luke's, Shoreditch, Finsbury, and Clerkenwell.

| No. of Beds. | No. of Beds Daily Occupied. | HOSPITALS. | In-patnts. | Out-patients. | Total Expenditure. | Income: Charitable. | Income: Proprietary. | Income: Patnts' pymnts. | Total Income. | Legacies not included in previous column. |
|---|---|---|---|---|---|---|---|---|---|---|
| | | | | | £ | £ | £ | £ | £ | £ |
| 96 | 78 | Metropolitan ... | 962 | 30,017 | 10,080 | 6,189 | 458 | 841 | 7,468 | 5,117 |
| 170 | 140 | Royal Free ... | 2,001 | 34,312 | 12,699 | 8,687 | 1,089 | ... | 9,776 | 4,063 |
| 80 | 57 | Royal, for Diseases of the Chest ... | 739 | 6,636 | 8,481 | 8,058 | 144 | ... | 8,202 | 545 |
| 57 | 48 | North-Eastern, for Children ... | 764 | 15,554 | 6,038 | 4,839 | 558 | 701 | 6,098 | ... |
| 36 | 25 | City of London Lying-In ... | 565 | 1,623 | 3,549 | 475 | 3,634 | 3 | 4,112 | 675 |
| 45 | 28 | St. Mark's, for Fistula ... | 349 | 995 | 3,748 | 5,796 | 637 | ... | 6,433 | 804 |
| 100 | 75 | Royal London Ophthalmic ... | 2,227 | 25,713 | 8,054 | 4,431 | 699 | ... | 5,130 | 2,888 |
| 45 | 21 | City Orthopædic ... | 140 | 2,202 | 2,703 | 2,611 | 53 | ... | 2,664 | ... |
| 16 | 12 | Central London Throat and Ear ... | 315 | 7,948 | 2,383 | 707 | 63 | 1,636 | 2,406 | 50 |
| 645 | 484 | DISPENSARIES. | 8,062 | 125,000 | 57,735 | 41,793 | 7,335 | 3,181 | 52,309 | 14,142 |
| ... | ... | City ... | ... | 4,980 | 1,359 | 1,015 | 214 | ... | 1,229 | ... |
| ... | ... | City of London and East London ... | ... | 20,090 | 1,481 | 95 | 59 | 1,696 | 1,850 | ... |
| ... | ... | Farringdon General ... | ... | 3,478 | 534 | 219 | ... | 204 | 423 | ... |
| ... | ... | Finsbury ... | ... | 12,239 | 868 | 697 | 150 | 229 | 1,076 | ... |
| ... | ... | Metropolitan ... | ... | 6,112 | 840 | 500 | 117 | 290 | 907 | ... |
| ... | ... | Royal General ... | ... | 3,023 | 880 | 564 | 347 | 95 | 1,006 | ... |
| 645 | 484 | | 8,062 | 174,922 | 63,697 | 44,883 | 8,222 | 5,695 | 58,800 | 14,142 |

### ST. MARYLEBONE AND WEST CENTRAL DISTRICT

Comprising St. Marylebone, St. John's Wood, Bloomsbury, Holborn, &c.

| No. of Beds. | No. of Beds Daily Occupied. | HOSPITALS. | In-patnts. | Out-patients. | Total Expenditure. | Income. Charitable. | Income. Proprietary | Income. Patnts' pymnts. | Total Income. | Legacies not included in previous column. |
|---|---|---|---|---|---|---|---|---|---|---|
| | | | | | £ | £ | £ | £ | £ | £ |
| 70 | 48 | French ... ... ... ... ... ... ... | 764 | 4,833 | 3,687 | 3,850 | 45 | ... | 3,895 | 3,210 |
| 5 | 3 | Italian ... ... ... ... ... ... ... | 95 | 4,583 | 770 | 2,629 | 118 | ... | 2,747 | ... |
| 100 | 78 | London Homœopathic ... ... ... ... | 1,111 | 18,551 | 12,131 | 3,225 | 2,895 | 762 | 6,882 | 2,632 |
| 24 | 12 | SS. John and Elizabeth ... ... ... | 26 | ... | 2,961 | 893 | 1,025 | ... | 1,918 | 909 |
| 372 | 304 | The Middlesex ... ... ... ... ... | 3,607 | 37,447 | 36,084 | 9,939 | 9,195 | ... | 19,134 | 12,081 |
| 68 | 34 | Alexandra for Children ... ... | 89 | 340 | 2,463 | 3,364 | 121 | 239 | 3,724 | ... |
| 30 | 28 | Hospital for Incurable Children ... ... | 34 | ... | 1,328 | 420 | 69 | 393 | 882 | 340 |
| 238 | 163 | Hospital for Sick Children ... ... ... | 2,305 | 24,249 | 17,534 | 5,379 | 3,626 | ... | 9,005 | 9,514 |
| 18 | 11 | British Lying-in ... ... ... ... | 269 | 411 | 2,674 | 474 | 1,669 | 73 | 2,216 | ... |
| 68 | 49 | Queen Charlotte's Lying-in ... ... ... | 1,223 | 1,070 | 4,554 | 3,751 | 276 | 141 | 4,168 | 3,060 |
| 42 | 38 | New Hospital for Women ... ... ... | 412 | 10,429 | 14,239 | 2,830 | 189 | 1,084 | 4,103 | 400 |
| 50 | 47 | Samaritan Free... ... ... ... ... | 542 | 8,093 | 6,296 | 4,479 | 208 | ... | 4,687 | 2,100 |
| 200 | 176 | National for the Paralysed, &c. ... ... | 1,030 | 5,969 | 14,868 | 4,742 | 1,750 | 1,842 | 8,334 | 9,708 |
| 25 | 16 | Hospital for Epilepsy, &c. ... ... ... | 88 | 781 | 1,676 | 696 | 40 | 490 | 1,226 | 950 |
| 50 | 26 | West End, for Epilepsy, &c.... ... ... | 196 | 3,511 | 2,769 | 2,196 | 30 | 495 | 2,721 | ... |
| 28 | 19 | Central London Ophthalmic ... ... ... | 310 | 11,060 | 1,586 | 1,183 | 12 | 229 | 1,424 | 100 |
| 13 | 5 | Western Ophthalmic ... ... ... ... | 132 | 7,498 | 665 | 427 | 133 | ... | 560 | ... |
| 60 | 53 | National Orthopædic ... ... ... ... | 212 | 758 | 2,447 | 1,151 | ... | 938 | 2,089 | 400 |
| 20 | 17 | Establishment for Gentlewomen ... ... | 152 | ... | 2,465 | 742 | 123 | 998 | 1,863 | 200 |
| ... | ... | National Dental ... ... ... ... | ... | 23,870 | 1,546 | 378 | ... | 1,076 | 1,454 | ... |
| 16 | 8 | London Throat ... ... ... ... ... | 465 | 2,815 | 1,381 | 489 | ... | 880 | 1,369 | ... |
| 4 | 1 | Metropolitan Ear, Nose, and Throat ... | 47 | 2,969 | 355 | 186 | ... | 213 | 399 | ... |
| 1,501 | 1,136 | DISPENSARIES. | 13,109 | 170,237 | 124,479 | 53,423 | 21,524 | 9,853 | 84,800 | 45,604 |
| ... | ... | Bloomsbury Provident ... ... ... ... | ... | 970 | 259 | 22 | ... | 203 | 225 | ... |
| ... | ... | London Medical Mission ... ... ... ... | ... | 6,881 | 1,305 | 839 | 70 | 266 | 1,175 | 225 |
| ... | ... | Portland Town ... ... ... ... ... | ... | 2,187 | 183 | 117 | 5 | 8 | 130 | 50 |
| ... | ... | Infirmary for Consumption ... ... ... | ... | 410 | 439 | 171 | 187 | ... | 358 | ... |
| ... | ... | St. John's Wood Provident ... ... ... | ... | 5,928 | 626 | 249 | 34 | 414 | 697 | ... |
| ... | ... | St. Marylebone General ... ... ... ... | ... | 2,845 | 771 | 430 | 178 | 222 | 830 | ... |
| ... | ... | Western General ... ... ... ... ... | ... | 14,359 | 1,343 | 1,029 | 31 | 44 | 1,104 | 95 |
| 1,501 | 1,136 | | 13,109 | 203,817 | 129,405 | 56,280 | 22,029 | 11,010 | 89,319 | 45,974 |

### KENSINGTON AND WEST DISTRICT.

Comprising Kensington, Paddington, Bayswater, Kilburn, Chelsea, Brompton, Fulham, Hammersmith, Chiswick, Brentford, Acton, Ealing, &c.

| No. of Beds. | No. of Beds Daily Occupied. | HOSPITALS. | In-patnts. | Out-patients. | Total Expenditure. | Income. Charitable. | Income. Proprietary | Income. Patnts' pymnts. | Total Income. | Legacies not included in previous column. |
|---|---|---|---|---|---|---|---|---|---|---|
| | | | | | £ | £ | £ | £ | £ | £ |
| 351 | 307 | St. George's ... ... ... ... ... ... | 4,006 | 24,521 | 43,219 | 9,220 | 15,977 | ... | 25,197 | 11,845 |
| 281 | 244 | St. Mary's ... ... ... ... ... ... | 3,710 | 36,816 | 25,589 | 9,816 | 2,097 | ... | 11,913 | 15,524 |
| 101 | 89 | West London ... ... ... ... ... ... | 1,518 | 31,170 | 7,418 | 5,978 | 222 | ... | 6,198 | 2,948 |
| 9 | 5 | Queen's Jubilee... ... ... ... ... ... | 114 | 4,661 | 1,000 | 1,113 | 21 | 15 | 1,149 | ... |
| 321 | 247 | Hospital for Consumption ... ... ... ... | 1,423 | 7,195 | 33,627 | 13,824 | 8,869 | ... | 22,693 | 22,132 |
| 24 | 13 | Belgrave, for Children ... ... ... ... | 262 | 5,263 | 1,426 | 794 | 96 | ... | 890 | ... |
| 50 | 50 | Cheyne, for Sick and Incurable Children ... | 67 | ... | 3,349 | 2,049 | 234 | 406 | 2,689 | 1,453 |
| 46 | 36 | Paddington Green, for Children ... ... | 601 | 14,070 | 3,473 | 2,636 | 148 | 302 | 3,086 | 4,400 |
| 110 | 83 | Victoria, for Children ... ... ... ... | 1,199 | 16,963 | 7,235 | 4,042 | 571 | 369 | 4,982 | 2,661 |
| 52 | 47 | Chelsea, for Women ... ... ... ... | 671 | 2,884 | 5,088 | 3,452 | 287 | 829 | 4,568 | 1,661 |
| 105 | 83 | Cancer ... ... ... ... ... ... ... | 835 | 1,642 | 12,223 | 3,962 | 2,765 | ... | 6,727 | 18,557 |
| 135 | 86 | Female Lock ... ... ... ... ... ... | 662 | ... | 4,109 | 2,263 | 7 | 1,371 | 3,641 | 650 |
| 16 | 10 | Epsom and Ewell Cottage ... ... ... | 123 | ... | 960 | 545 | 11 | 210 | 766 | 100 |
| 25 | 13 | Reigate and Redhill Cottage... ... ... | 190 | ... | 2,113 | 1,676 | 52 | 109 | 1,837 | 500 |
| 16 | 7 | Wimbledon Cottage ... ... ... ... | 112 | ... | 585 | 377 | 5 | 125 | 507 | ... |
| 15 | 7 | Hounslow Cottage ... ... ... ... | 97 | 598 | 467 | 267 | 196 | 56 | 519 | 437 |
| 1,657 | 1,327 | DISPENSARIES. | 15,590 | 155,783 | 151,881 | 62,012 | 31,558 | 3,792 | 97,362 | 82,868 |
| ... | ... | Brompton Provident ... ... ... ... | ... | 979 | 468 | 127 | 63 | 237 | 427 | ... |
| ... | ... | Chelsea, &c. ... ... ... ... ... | ... | 4,372 | 523 | 301 | 136 | ... | 437 | 153 |
| ... | ... | Chelsea Provident ... ... ... ... | ... | 991 | 300 | 19 | ... | 240 | 259 | ... |
| ... | ... | Kensal Town Provident ... ... ... ... | ... | 726 | 327 | 19 | ... | 298 | 317 | ... |
| ... | ... | Kensington ... ... ... ... ... | ... | 4,091 | 602 | 535 | 49 | ... | 584 | ... |
| ... | ... | Kilburn, Maida Vale ... ... ... ... | ... | 2,048 | 515 | 411 | 47 | ... | 458 | ... |
| ... | ... | Kilburn Provident ... ... ... ... | ... | 5,042 | 1,153 | 63 | 9 | 1,082 | 1,154 | ... |
| ... | ... | Notting Hill Provident ... ... ... ... | ... | 351 | 192 | 63 | 5 | 78 | 146 | ... |
| ... | ... | Paddington Provident... ... ... ... | ... | 3,377 | 539 | 148 | 26 | 363 | 537 | ... |
| ... | ... | Pimlico Provident ... ... ... ... | ... | 916 | 713 | 11 | ... | 733 | 744 | ... |
| ... | ... | Royal Pimlico Provident ... ... ... | ... | 5,064 | 1,193 | 416 | 31 | 560 | 1,007 | ... |
| ... | ... | Westbourne Provident ... ... ... ... | ... | 1,029 | 454 | 37 | 43 | 319 | 399 | ... |
| 1,657 | 1,327 | | 15,590 | 184,769 | 158,860 | 64,162 | 31,967 | 7,702 | 103,831 | 83,021 |

### ISLINGTON AND NORTH-WEST DISTRICT.

Comprising Islington, Holloway, Highbury, Hampstead, Highgate, St. Pancras, Stoke Newington, Tottenham, &c.

| No. of Beds. | No. of Beds Daily Occupied. | Hospitals. | In-patnts. | Out-patients. | Total Expenditure. | Income. Charitable. | Income. Proprietary. | Income. Patnts' pymnts. | Total Income. | Legacies not included in previous column. |
|---|---|---|---|---|---|---|---|---|---|---|
| | | | | | £ | £ | £ | £ | £ | £ |
| 113 | 108 | Great Northern Central ... ... ... | 1,701 | 28,160 | 10,430 | 6,547 | 974 | 569 | 8,090 | 7,550 |
| 31 | 21 | Hampstead Hospital ... ... ... ... | 327 | 840 | 3,518 | 2,556 | 465 | 355 | 3,376 | ... |
| 120 | 72 | London Temperance ... ... ... ... | 1,290 | 18,731 | 11,063 | 6,378 | 2,947 | 359 | 9,684 | 305 |
| 52 | 46 | North-West London ... ... ... ... | 601 | 18,033 | 4,285 | 3,826 | 318 | 36 | 4,180 | 100 |
| 103 | 29 | Tottenham Training ... ... ... ... | 297 | 8,081 | 3,902 | 2,146 | 364 | 200 | 2,710 | 39 |
| 185 | 158 | University College ... ... ... ... | 2,922 | 46,846 | 18,076 | 11,368 | 3,015 | 45 | 14,428 | 8,060 |
| 60 | 55 | North London Consumption ... ... ... | 460 | 3,502 | 5,976 | 6,695 | 118 | 56 | 6,869 | 60 |
| 150 | 60 | London Fever ... ... ... ... ... | 599 | ... | 12,682 | 7,624 | 1,661 | 1,679 | 10,964 | 3,112 |
| 28 | 19 | Invalid Asylum ... ... ... ... ... | 193 | ... | 1,011 | 712 | 136 | 271 | 1,119 | 25 |
| 16 | 14 | Children's Home Hospital, Barnet ... ... | 69 | ... | 564 | 365 | 105 | 84 | 554 | 10 |
| 14 | 8 | Enfield Cottage ... ... ... ... ... | 98 | ... | 1,704 | 1,486 | 66 | 33 | 1,585 | ... |
| 30 | 25 | Memorial Cottage, Mildmay ... ... ... | 207 | ... | 1,664 | 462 | 974 | 134 | 1,570 | 14 |
| 28 | 13 | St. Saviour's Home ... ... ... ... | 90 | ... | 1,991 | 1,164 | 6 | 599 | 1,769 | 25 |
| 42 | 32 | Friedenheim Home ... ... ... ... | 105 | ... | 3,625 | 3,809 | 73 | 212 | 4,094 | 1,500 |
| 33 | 23 | St. Monica's, Brondesbury ... ... ... | 51 | ... | 1,522 | 726 | 172 | 235 | 1,133 | 700 |
| 1,005 | 683 | DISPENSARIES. | 9,010 | 124,193 | 82,013 | 55,864 | 11,394 | 4,877 | 72,135 | 21,500 |
| ... | ... | Child's Hill Provident ... ... ... ... | ... | 1,000 | 329 | 38 | 13 | 278 | 329 | ... |
| ... | ... | Camden Provident ... ... ... ... | ... | 991 | 300 | 19 | ... | 242 | 261 | ... |
| ... | ... | Hampstead Provident ... ... ... ... | ... | 11,141 | 1,025 | 241 | 34 | 759 | 1,034 | ... |
| ... | ... | Holloway and North Islington ... ... | ... | 3,725 | 769 | 293 | 47 | 345 | 685 | ... |
| ... | ... | Islington ... ... ... ... ... ... | ... | 12,239 | 887 | 286 | 19 | 566 | 871 | ... |
| ... | ... | St. Pancras and Northern ... ... ... | ... | 1,619 | 529 | 235 | 133 | 109 | 477 | 500 |
| ... | ... | Stamford Hill, &c. ... ... ... ... | ... | 5,382 | 823 | 518 | 165 | ... | 683 | ... |
| 1,005 | 683 | | 9,010 | 160,290 | 86,675 | 57,494 | 11,805 | 7,176 | 76,475 | 22,000 |

### WESTMINSTER DISTRICT.

Comprising Westminster City and Liberties.

| No. of Beds. | No. of Beds Daily Occupied. | Hospitals. | In-patnts. | Out-patients. | Total Expenditure. | Charitable. | Proprietary. | Patnts' pymnts. | Total Income. | Legacies. |
|---|---|---|---|---|---|---|---|---|---|---|
| | | | | | £ | £ | £ | £ | £ | £ |
| 175 | 141 | Charing Cross ... ... ... ... ... | 2,155 | 19,616 | 15,600 | 12,251 | 1,609 | 166 | 14,026 | 10,120 |
| 217 | 179 | King's College ... ... ... ... ... | 2,384 | 23,930 | 18,457 | 9,619 | 2,586 | 180 | 12,385 | 5,101 |
| 202 | 179 | Westminster ... ... ... ... ... | 2,637 | 24,217 | 17,166 | 5,388 | 2,951 | 89 | 8,428 | 5,393 |
| 133 | 120 | Ventnor, for Consumption ... ... ... | 711 | ... | 18,967 | 5,652 | 2,321 | 3,441 | 11,414 | 10,957 |
| 20 | 15 | Grosvenor, for Women and Children ... | 191 | 2,696 | 1,924 | 1,062 | 7 | 487 | 1,556 | 146 |
| 61 | 47 | Hospital for Women ... ... ... ... | 728 | 5,002 | 6,263 | 3,715 | 304 | 582 | 4,601 | 780 |
| 25 | 21 | National, for Diseases of Heart, &c... ... | 178 | 1,974 | 2,351 | 1,762 | 58 | 436 | 2,256 | 1,020 |
| 36 | 26 | Royal Westminster Ophthalmic ... ... | 568 | 10,186 | 2,171 | 1,356 | 582 | 68 | 2,006 | 2,837 |
| 50 | 46 | Royal Orthopædic ... ... ... ... | 180 | 726 | 2,079 | 755 | 490 | 182 | 1,427 | 2,143 |
| 9 | 8 | Royal Ear ... ... ... ... ... | 251 | 2,068 | 785 | 227 | ... | 616 | 843 | ... |
| ... | ... | Dental ... ... ... ... ... ... | ... | 33,294 | 2,039 | 2,589 | 246 | 148 | 2,983 | 110 |
| 20 | 13 | Gordon, for Fistula ... ... ... ... | 221 | 945 | 1,358 | 441 | ... | 815 | 1,256 | ... |
| 27 | 20 | St. Peter's, for Stone ... ... ... ... | 502 | 4,773 | 3,533 | 1,051 | 367 | 2,095 | 3,513 | ... |
| 975 | 815 | DISPENSARIES. | 10,706 | 129,427 | 92,693 | 45,868 | 11,521 | 9,305 | 66,694 | 38,607 |
| ... | ... | Public ... ... ... ... ... ... | ... | 3,315 | 740 | 501 | 170 | ... | 671 | 50 |
| ... | ... | St. George and St. James ... ... ... | ... | 2,043 | 625 | 369 | 14 | 28 | 411 | ... |
| ... | ... | St. George's, Hanover Square ... ... | ... | 776 | 523 | 400 | ... | 125 | 525 | ... |
| ... | ... | Western ... ... ... ... ... ... | ... | 10,671 | 1,495 | 353 | 460 | 653 | 1,466 | ... |
| ... | ... | Westminster General ... ... ... ... | ... | 6,853 | 603 | 405 | 164 | 111 | 680 | ... |
| 975 | 815 | | 10,706 | 153,085 | 96,679 | 47,896 | 12,329 | 10,222 | 70,447 | 38,657 |

### STRATFORD AND EAST END DISTRICT.

Comprising Bethnal Green, Tower Hamlets, West Ham, Whitechapel, Hackney, Stepney, Limehouse, Poplar, and the East.

| No. of Beds. | No. of Beds Daily Occupied. | Hospitals. | In-patnts. | Out-patients. | Total Expenditure. | Charitable. | Proprietary. | Patnts' pymnts. | Total Income. | Legacies. |
|---|---|---|---|---|---|---|---|---|---|---|
| 130 | 123 | German ... ... ... ... ... ... | 1,733 | 24,079 | 13,604 | 10,139 | 2,257 | 524 | 12,920 | 1,050 |
| 776 | 656 | London ... ... ... ... ... ... | 11,009 | 178,838 | 89,379 | 96,737 | 24,352 | 597 | 121,686 | 10,776 |
| 65 | 51 | Poplar ... ... ... ... ... ... | 993 | 20,194 | 9,655 | 7,705 | 763 | 124 | 8,582 | 1,850 |
| 60 | 39 | West Ham, &c. ... ... ... ... ... | 707 | 21,006 | 4,696 | 4,058 | 212 | ... | 4,270 | ... |
| 34 | 22 | Walthamstow, &c. ... ... ... ... | 391 | 730 | 999 | 1,515 | 233 | ... | 1,748 | ... |
| 164 | 74 | City of London for Diseases of the Chest ... | 596 | 9,870 | 8,638 | 11,762 | 167 | ... | 11,929 | 693 |
| 130 | 91 | East London for Children ... ... ... | 1,552 | 34,456 | 8,247 | 7,244 | 1,024 | ... | 8,268 | 1,775 |
| 92 | 40 | Mrs. Gladstone's Home ... ... ... | 597 | ... | 877 | 459 | 371 | ... | 830 | ... |
| 18 | 10 | East End Mothers' Home ... ... ... | 255 | 293 | 1,767 | 1,007 | 498 | 53 | 1,558 | 100 |
| 50 | 37 | Mildmay Mission Hospital ... ... ... | 452 | 6,814 | 3,324 | 2,189 | 541 | 42 | 2,772 | 250 |
| 38 | 35 | St. Mary's, Plaistow ... ... ... ... | 631 | 10,094 | 2,773 | 2,377 | 163 | 75 | 2,615 | ... |
| 8 | 6 | Passmore Edwards Cottage, Tilbury ... | 69 | 365 | 625 | 790 | 8 | ... | 798 | 20 |
| 1,565 | 1,184 | DISPENSARIES. | 18,985 | 306,739 | 144,584 | 145982 | 30,579 | 1,415 | 177,976 | 16,514 |
| ... | ... | Eastern ... ... ... ... ... ... | ... | 5,711 | 699 | 290 | 278 | 72 | 640 | 100 |
| ... | ... | Hackney Provident ... ... ... ... | ... | 864 | 228 | 48 | ... | 186 | 234 | ... |
| ... | ... | London ... ... ... ... ... ... | ... | 2,303 | 601 | 136 | 268 | ... | 404 | 1,000 |
| ... | ... | Queen Adelaide's ... ... ... ... | ... | 6,338 | 476 | 296 | 211 | 30 | 537 | 180 |
| ... | ... | Tower Hamlets ... ... ... ... ... | ... | 3,567 | 560 | 310 | 26 | 144 | 480 | ... |
| ... | ... | Whitechapel Provident ... ... ... | ... | 5,161 | 824 | 15 | ... | 842 | 857 | ... |
| 1,565 | 1,184 | | 18,985 | 330,683 | 147,972 | 147077 | 31,362 | 2,689 | 181,128 | 17,794 |

### THE METROPOLITAN HOSPITALS.—A SUMMARY OF THE WORK DONE IN 1898.

It will be seen from the following summary that the Voluntary Hospitals and Medical Charities of London, during the twelve months ending 31st December, 1898, relieved over One million six hundred thousand patients at a cost of £868,693. The Ordinary Income only amounted to £742,902, leaving a deficiency of £125,791 on the year's work. The Legacies received in 1898 amounted to £262,441, being £69,023 more than the amount received in 1897.

| No. of Beds. | No. of Beds Daily Occupied. | HOSPITALS AND DISPENSARIES. | In-patients. | Out-patients. | Total Expenditure. | Income. | | | Total Income. | Legacies not included in previous column. |
|---|---|---|---|---|---|---|---|---|---|---|
| | | | | | | Charitable. | Proprietary. | Patients' Payments. | | |
| | | | | | £ | £ | £ | £ | £ | £ |
| 2,314 | 1,585 | Newington and South District ... | 22,970 | 299,990 | 185,405 | 64,678 | 82,868 | 15,356 | 162,902 | 40,853 |
| 645 | 484 | City and East Central District ... | 8,062 | 174,922 | 63,697 | 44,883 | 8,222 | 5,695 | 58,800 | 14,142 |
| 975 | 815 | Westminster District ... ... | 10,706 | 153,085 | 96,679 | 47,896 | 12,329 | 10,222 | 70,447 | 38,657 |
| 1,501 | 1,136 | St. Marylebone and West Central District ... ... ... ... | 13,109 | 203,817 | 129,405 | 56,280 | 22,029 | 11,010 | 89,319 | 45,974 |
| 1,657 | 1,327 | Kensington and West District ... | 15,590 | 184,769 | 158,860 | 64,162 | 31,967 | 7,702 | 103,831 | 83,021 |
| 1,005 | 683 | Islington and North-West District | 9,010 | 160,290 | 86,675 | 57,494 | 11,805 | 7,176 | 76,475 | 22,000 |
| 1,565 | 1,184 | Stratford and East-End District... | 18,985 | 330,683 | 147,972 | 147,077 | 31,362 | 2,689 | 181,128 | 17,794 |
| 9,662 | 7,214 | | 98,432 | 1,507,556 | 868,693 | 482,470 | 200,582 | 59,850 | 742,902 | 262,441 |

### SPECIAL HOSPITALS—(*continued*).

### THROAT AND EAR.

Among the patients attending hospitals for the throat and ear are sufferers from ear-ache, leading to the loss of the precious sense of hearing; employés threatened with the loss of work and wages on account of deafness; children handicapped in the race for life with defective hearing; victims of "running from the ear," which, if not attended to, often results in abscess of the brain and death; and individuals troubled by an obstruction in the nose, such as polypus. The misery resulting to people who, from any defect in the nasal passage, become the chronic subjects of "mouth breathing," with injurious results not merely to the functions of speech and audition, but also to more distinct though not less serious reflexes on their nervous system and general health, is but faintly grasped by the public. Not only diseases of the throat and ear, but also of the nose are treated in the hospital which, founded in 1874 in a central part of the town, has rapidly extended its area of usefulness. In the first five years of its existence the number of new patients was 19,300, involving 72,600 separate attendances; while in the last five—from the year 1893 to the year 1898—the numbers were 38,398 and 228,232 respectively.

The oldest institution in Europe connected specially with diseases of the ear and nose, claims to have relieved and cured annually since 1816 thousands of acute and severe cases of this class of disease. In the past year, in addition to the very large numbers who are daily treated as out-patients, upwards of 300 cases have been operated upon as in-patients in the wards, and thus rescued from otherwise inevitable permanent deafness, and placed in a condition of hearing enabling them to gain their own livelihood comfortably. A new building has been badly needed for some time, but the funds in hand are yet inadequate; and, although the site has been purchased, the committee will not feel justified in proceeding further until about £3,000 more has been subscribed, though the existing building is not adapted to the successful treatment of severe cases. The new hospital has been planned with a view to introduce every modern improvement that sanitary and surgical science requires.

### WOMEN'S.

A hospital founded 27 years ago with ten beds for the benefit of women, has been three times enlarged. The first enlargement was in 1875, and in 1890 it was rebuilt. The present hospital contains 52 beds, and a large and suitable out-patient department. Again, in 1898 five beds for cancer cases were added, also a separate ward for the patients requiring treatment for the eyes. At the same time the old quarters of the nurses were pulled down and a new home erected. Still the number of patients continues to increase, and there are always 30 or 40 waiting their turn for admission. The in-patients are expected to pay half-a-crown a week towards their maintenance, but this is often remitted in the case of very poor women, and no suitable case is rejected on account of poverty. Unhappily, the authorities are daily obliged to send away numbers of new patients, as it is simply impossible to treat all who seek advice. Many of these women live at a long distance, and bring their children as patients. The committee, in order to meet this state of things, contemplate opening a children's department at another hour of the day, the mothers being evidently extremely anxious to have their children under the same care. There is a maternity department for the treatment of poor women in their own homes, and a fully qualified medical woman is occupied entirely in this work. The new buildings are all paid for, but there is an annual deficit of about £2,500 to be met by contributions from the public.

A great feature at one of the hospitals for the exclusive benefit of women is the number of patients who arrive at its doors in a critical condition. Yet the rate of mortality in the hospital stands at only one and four-fifths per cent., a fact which speaks more eloquently than any words could do of the skill and attention shown by the doctors and nurses. At this hospital excellent accommodation for the nurses has lately been provided at an outlay of nearly £2,000. There is also a convalescent home to which last year 249 patients were admitted.

